# Association of visceral fat area with early-stage locomotive syndrome across various age groups: a cross-sectional study

**DOI:** 10.1038/s41598-024-76478-8

**Published:** 2024-10-26

**Authors:** Tadashi Miyazaki, Naoki Ozato, Tohru Yamaguchi, Yoko Sugiura, Hiromitsu Kawada, Yoshihisa Katsuragi, Noriko Osaki, Tatsuya Mikami, Ken Ito, Koichi Murashita, Shigeyuki Nakaji, Yoshinori Tamada

**Affiliations:** 1 Department of Medical Data Intelligence, Research Center for Health-Medical Data Science, Graduate School of Medicine, Hirosaki University, Aomori, Japan; 2 Human Health Care Products Research Laboratories, Kao Corporation, Tokyo, Japan; 3 Research and Development, Kao Corporation, Tokyo, Japan; 4 Department of Active Life Promotion Sciences, Graduate School of Medicine, Hirosaki University, Aomori, Japan; 5 Department of Preemptive Medicine, Research Innovation Center for Health Promotion, Graduate School of Medicine, Hirosaki University, Aomori, Japan; 6 Department of Stress Response Science, Biomedical Research Center, Graduate School of Medicine, Hirosaki University, Aomori, Japan; 7 Research Institute of Health Innovation, Graduate School of Medicine, Hirosaki University, Aomori, Japan; 8 Department of Social Medicine, Graduate School of Medicine, Hirosaki University, Aomori, Japan

**Keywords:** Visceral fat area, Locomotive syndrome, Metabolic syndrome, Cross-sectional study, Health care, Medical research

## Abstract

The association between visceral fat area (VFA) and locomotive syndrome (LS) has been extensively studied in the older population; however, the association between VFA and early-stage LS (stage 1 [LS1]) remains unclear. In this cross-sectional study, we investigated this association across different age groups. The study involved 1,236 (524 male and 712 female) participants (aged 20–85 years). Multiple regression analysis adjusted for sex, body mass index, skeletal muscle mass index, T-score, exercise habits, smoking status, and alcohol consumption revealed a significant association between LS1 and VFA across all VFA quartiles. The adjusted odds ratio OR for quartiles 2, 3, and 4 was 1.84, 2.68, and 4.12, respectively. The association between LS1 and VFA across the age groups—high VFA (> 73 cm^2^) and non-older (< 65 years) (OR, 1.87; 95% CI, 1.28–2.72; *p* = 0.001), low VFA (≤ 73 cm^2^) and older (≥ 65 years) (OR, 3.16; 95% CI, 1.94–5.14; *p* < 0.001), and high VFA and older groups (OR, 6.43; 95% CI, 3.98–10.4; *p* < 0.001)—was significantly stronger than that in the low VFA and non-older group. In summary, our findings suggest that managing VFA through diet and exercise is crucial for preventing LS1 across all age groups.

## Introduction

In Japan, comprising a super-aged society, the population aged ≥ 65 years reached 36.24 million, accounting for 29.0% of the total population in 2022^1^. Approximately 18.9% of individuals in this age group require nursing care or support^2^, with one-quarter of all nursing care needs stemming from a decline in motor function^3^.

Locomotive syndrome (LS) is defined as a condition of decreased mobility due to impaired motor organs. It is categorised into no LS risk (non-LS), stage 1 (LS1), stage 2 (LS2), and stage 3 (LS3) according to the LS risk test by the Japanese Orthopaedic Association (JOA)^4–6^. Previous studies have defined LS as LS1 or higher or LS2 or higher and reported associations between LS and body composition^7–12^, physical performance^13–17^, lifestyle habits^8,18,19^, and musculoskeletal diseases^20–23^. However, studies focusing on the risk factors associated with LS1 are limited. According to the Research on Osteoarthritis/Osteoporosis Against Disability (ROAD) study, the prevalence of LS1 begins to increase at a younger age compared to that of LS2 and LS3^24,25^. Specifically, LS1 has the highest prevalence rate (22%–55% for male individuals and 25%–44% for female individuals) among other locomotive stages in age groups ranging from 20 to 70 s. Additionally, a longitudinal study comparing the physical performance in non-LS and LS1 groups suggested that the prevention of LS1 is important for future motor performance^26^. Together, these studies suggest that focusing on LS1 and determining the factors related to LS1 targeting a wide range of age groups are essential to prevent LS.

Obesity is one of the contributing factors to LS. Several studies have focused on obesity-related factors, such as body mass index (BMI)^7–9^, percent body fat (pBF)^10^, and waist circumference (WC)^11,12^, while only one study specifically addressed the visceral fat area (VFA), a key factor for metabolic syndrome (MetS)^27^. A previous study targeting a specific age group (≥ 85 years) reported that compared to BMI, pBF, and WC, VFA showed a better discriminative performance for mobility disability equivalent to LS3, as evaluated using a loco-check questionnaire^28^. Studies have reported an increased prevalence of LS1 at a young age^25^; furthermore, VFA increases with age^29^. Nevertheless, the involvement of VFA in LS1 remains unclear. Moreover, to the best of our knowledge, studies focusing on the association between LS1 and VFA in a wide range of age groups are scant. Therefore, stratifying VFA by age group and investigating their associations is essential to clarify the association between LS1 and VFA.

In this study, we aimed to investigate the association between LS1 and VFA and to clarify the involvement of VFA in LS1 across different age groups through a cross-sectional analysis. The findings of this study may be clinically important to prevent LS through VFA management and determine the association between LS and MetS.

## Methods

### Study design

**Fig. 1:**
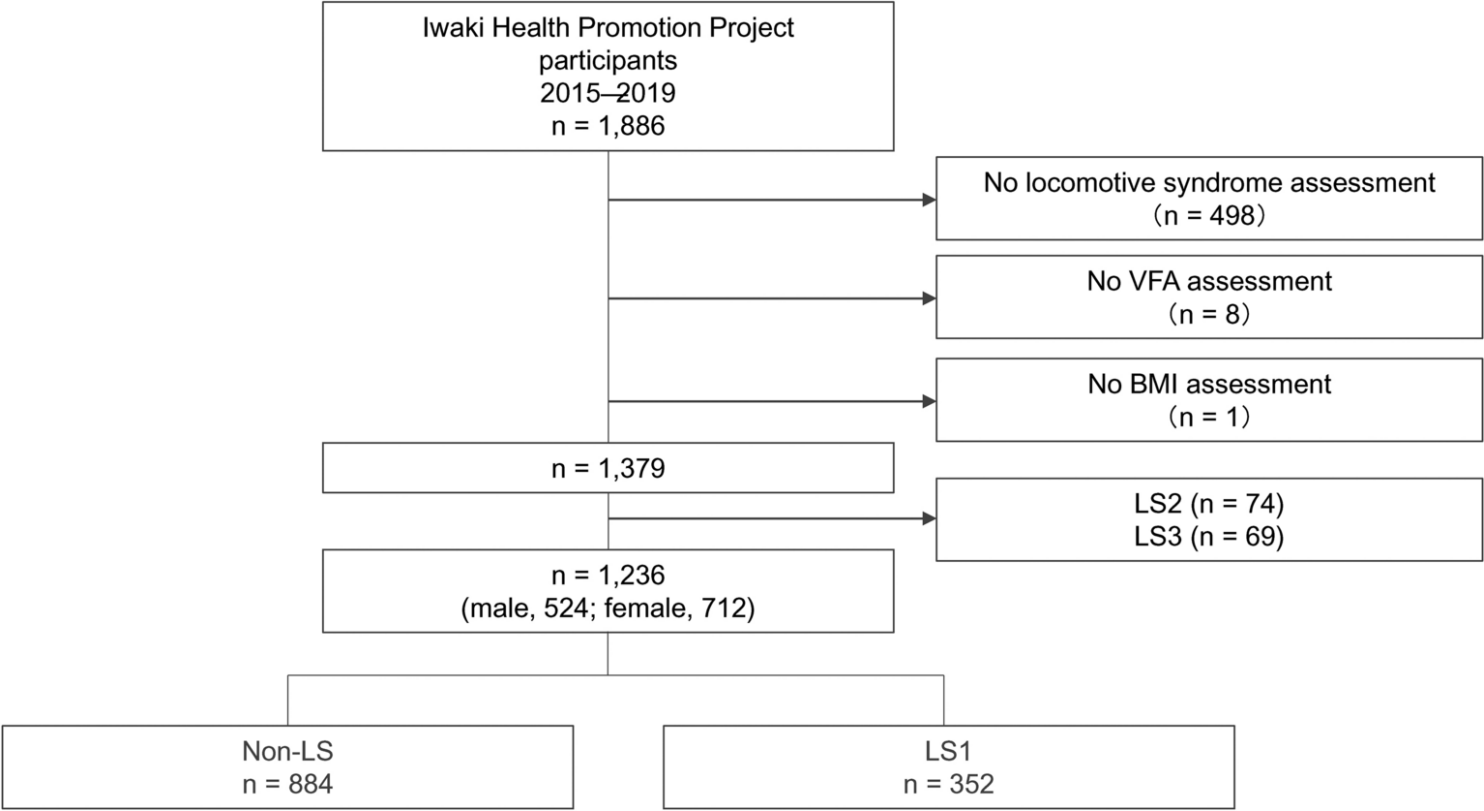
Flowchart of the study enrolment process. LS1, LOCOMO stage 1; LS2, LOCOMO stage 2; LS3, LOCOMO stage 3.

In this cross-sectional study, we used data obtained from annual health checkups as part of the Iwaki Health Promotion Project launched in 2005^30^. The participants were adults aged ≥ 20 years who lived in the Iwaki Region of Hirosaki City, Aomori Prefecture, Japan. This study focused on the association between LS1 and VFA in 1,886 individuals who underwent health checkups between 2015 and 2019. The analysis included participants who underwent a health checkup in 2015 and those who participated for the first time from 2016 to 2019, in chronological order without duplication starting in 2016. After excluding those with missing clinical data (no LS assessment, n = 498; no VFA assessment, n = 8; no BMI assessment, n = 1) and those with LS2 (n = 74) or LS3 (n = 69) status, 1,236 (524 male and 712 female) participants (age, 20–85 years) were finally included in the analysis (Fig. 1).

This study was approved by the Ethics Committee of Hirosaki University School of Medicine and was conducted in accordance with the principles of the Declaration of Helsinki (2014-377, 2016-028, 2017-026, 2018-012, 2018-063, and 2019-009). Written informed consent was obtained from all participants before inclusion in this study.

### Locomotive syndrome risk test

To classify LS severity, we used the stand-up test, two-step test, and 25-question Geriatric locomotive function scale (GLFS-25)^31,32^. The stand-up test was conducted to assess vertical mobility. The participants were instructed to stand up from four seats of different heights (40, 30, 20, and 10 cm) in a descending order of height, first with both legs and then with one leg. The difficulty levels were defined as 40 < 30 < 20 < 10 cm for both legs and 40 < 30 < 20 < 10 cm for one leg. The minimum height from which the participants could stand up and maintain their posture for 3 s was recorded. The two-step test was conducted to assess horizontal mobility. The participants were instructed to take two steps for as long as possible, and the longest distance was recorded as the length of the two steps (cm). The two-step test score was calculated using the following formula: length of the two steps (cm) / height (cm). GLFS-25 is a self-reported questionnaire comprising 25 items graded on a 5-point scale, from 0 (no impairment) to 4 (severe impairment). The GLFS-25 score is the sum of scores of each item, which ranged from 0 to 100 points.

### Diagnosis of locomotive syndrome

The stages of LS (non-LS and LS1) were determined using the clinical levels devised by the JOA. The participants who met one of the following criteria were diagnosed with LS1:

1. Stand-up test: The participants could not stand up from the 40-cm seat using either leg but could stand up from the 20-cm seat with both legs.
2. Two-step test: 1.1 ≤ two-step test score (cm/cm) < 1.3.
3. GLFS-25: 7 ≤ GFLS-25 score (points) < 16.

### Visceral fat area

VFA was measured using a bioimpedance-type visceral fat metre (EW-FA90; Panasonic Corporation, Osaka, Japan), certified as a medical device in Japan (No. 22500BZX00522000). The results obtained using this device correlated with those of computed tomography, the gold standard method for VFA measurement^33^.

### Other parameters

Body weight and skeletal muscle mass were measured using bioelectrical impedance analysis (Tanita MC-190 body composition analyser; Tanita Co., Ltd., Tokyo, Japan). BMI (kg/m^2^) was calculated as body weight (kg) divided by height squared (m) and the participants were classified into underweight (BMI < 18.5), normal (18.5 ≤ BMI < 25), and overweight (25 ≤ BMI) categories according to the criteria of the Japan Society for the Study of Obesity^34^. The skeletal muscle mass index (SMI) was calculated as skeletal muscle mass (kg) divided by height squared (m). Bone status was evaluated using the osteo-sonographic assessment index (OSI) T-score. The OSI was calculated using the speed of sound (SOS) and transmission index (TI) measured using a quantitative ultrasound device (AOS-100NW; ALOKA Co., Ltd., Tokyo, Japan) at the calcaneus of the foot. The OSI and T-score were calculated using the following formulae:

$${\text{OSI}}\, = \,{\text{TI}}\, \times \,{\text{SOS}}^{2} .$$

$${\text{T - score }}\,\left( \% \right)\, = \,({\text{Measured OSI/mean of OSI in young adults)}}\, \times \,100.$$

Lifestyle habits and physical activity were assessed using self-administered questionnaires and converted into binary categorical variables. For the smoking status, participants were provided with three options—non-smoker, current smoker, or former smoker—and those who chose non-smoker or former smoker were classified as ‘none,’ whereas those who chose current smoker were classified as ‘smoker.’ Similarly, for alcohol consumption, participants reported their drinking status over the past year as none, current drinker, or former drinker. Those who answered none or former drinker were classified as ‘none,’ and those who answered current drinker were classified as ‘current drinker.’ For physical activity, participants were asked whether they engage in regular exercise or sports at least once a week during seasons other than winter and were provided with two options—yes and no—and grouped accordingly.

The explanatory variables used in the later-described regression model included age, BMI (< 18.5, 18.5 ≤ BMI < 25, 25 ≤), SMI, T-score, exercise habits (none or ≥ 1 day/week in non-winter seasons), smoking status (none or current smoker), and alcohol consumption (none or current drinker).

### Statistical analyses

The participants were classified into the non-LS and LS1 groups and the following statistical analyses were performed between the groups. Differences in the continuous variables (VFA, age, height, weight, BMI, skeletal muscle mass, and SMI) between the non-LS and LS1 groups were assessed using the Wilcoxon rank-sum test; they are reported as mean ± standard deviation. Differences in proportions among categorical variables (sex, exercise habits, smoking status, and alcohol consumption) between the non-LS and LS1 groups were evaluated using the Chi-square test and reported as percentage.

The Cochran–Armitage trend test was conducted to assess the trend in the proportions of LS1 prevalence among VFA quartiles (quartile 1: VFA ≤ 45 cm^2^, n = 311; quartile 2: 45 cm^2^ < VFA ≤ 73 cm^2^, n = 309; quartile 3: 73 cm^2^ < VFA ≤ 104 cm^2^, n = 310; quartile 4: 104 cm^2^ < VFA, n = 306) and age groups (young adult group, 20 ≤ age < 40 years; middle-aged group, 40 ≤ age < 65 years; and older group, 65 years ≤ age).

Multiple logistic regression analysis was performed to examine the association between LS1 prevalence, VFA, and age group. The presence or absence of LS1 was entered into the regression model as the dependent variable; the VFA quartiles or age groups were considered independent variables; and sex, BMI, SMI, T-score, exercise habits, smoking status, and alcohol consumption as adjusted variables in the model.

A multiple logistic regression analysis was performed to investigate the association between LS1 prevalence and four groups stratified by median VFA (low VFA, ≤ 73 cm^2^; high VFA, > 73 cm^2^) and age groups (non-older group, < 65 years; older group, > 65 years): low VFA and non-older (n = 525), high VFA and non-older (n = 488), low VFA and older (n = 95), and high VFA and older (n = 128) groups. The presence or absence of LS1 was used as the dependent variable in the regression model. We included the four groups (reference: low VFA and non-older group) as independent variables, along with sex, BMI, SMI, T-score, exercise habits, smoking status, and alcohol consumption as adjusting variables in the model. Adjusted odds ratio (OR) and 95% confidence intervals (CI) were calculated. Statistical tests were two-tailed, and results with a *p*-value of < 0.05 were considered significant. All statistical analyses were performed using the R software version 4.3.0.

## Results

This study involved 1,236 (524 male and 712 female) participants aged 20–85 years categorised into the non-LS (n = 884, 71.5%) and LS1 (n = 352, 28.5%) groups (Fig. 1). The characteristics of the non-LS and LS1 groups are summarised in Table 1.

**Table 1:** Participant characteristics.

	Non-LS	LS1	*p*
N	884	352	
VFA (mean [SD])	75.5 (42.8)	90.8 (45.2)	< 0.001
Age (mean [SD])	45.3 (14.4)	54.9 (15.3)	< 0.001
20 s	129 (14.6)	20 (5.7)	
30 s	240 (27.1)	49 (13.9)	
40 s	177 (20.0)	66 (18.8)	
50 s	141 (16.0)	52 (14.8)	
60 s	161 (18.2)	99 (28.1)	
70 s	36 (4.1)	58 (16.5)	
80 s	0	8 (2.3)	
Sex = female (%)	498 (56.3)	214 (60.8)	0.171
Height (mean [SD])	163.3 (8.7)	160.9 (9.2)	< 0.001
Weight (mean [SD])	60.0 (12.1)	61.3 (12.8)	0.129
BMI (mean [SD])	22.4 (3.3)	23.6 (3.7)	< 0.001
Skeletal muscle mass (mean [SD])	42.9 (9.2)	41.9 (8.9)	0.109
SMI (mean [SD])	15.9 (2.1)	16.0 (2.0)	0.190
Exercise habit = none (%)	547 (61.9)	225 (63.9)	0.546
Smoking status = none (%)	710 (80.3)	282 (80.1)	0.999
Alcohol consumption = none (%)	459 (51.9)	195 (55.4)	0.298

Continuous variables are presented as mean (SD). Categorical variables are presented as N (%).

*SD* Standard deviation, *VFA* Visceral fat area, *BMI* Body mass index, *SMI* Skeletal muscle mass index.

**Fig. 2:**
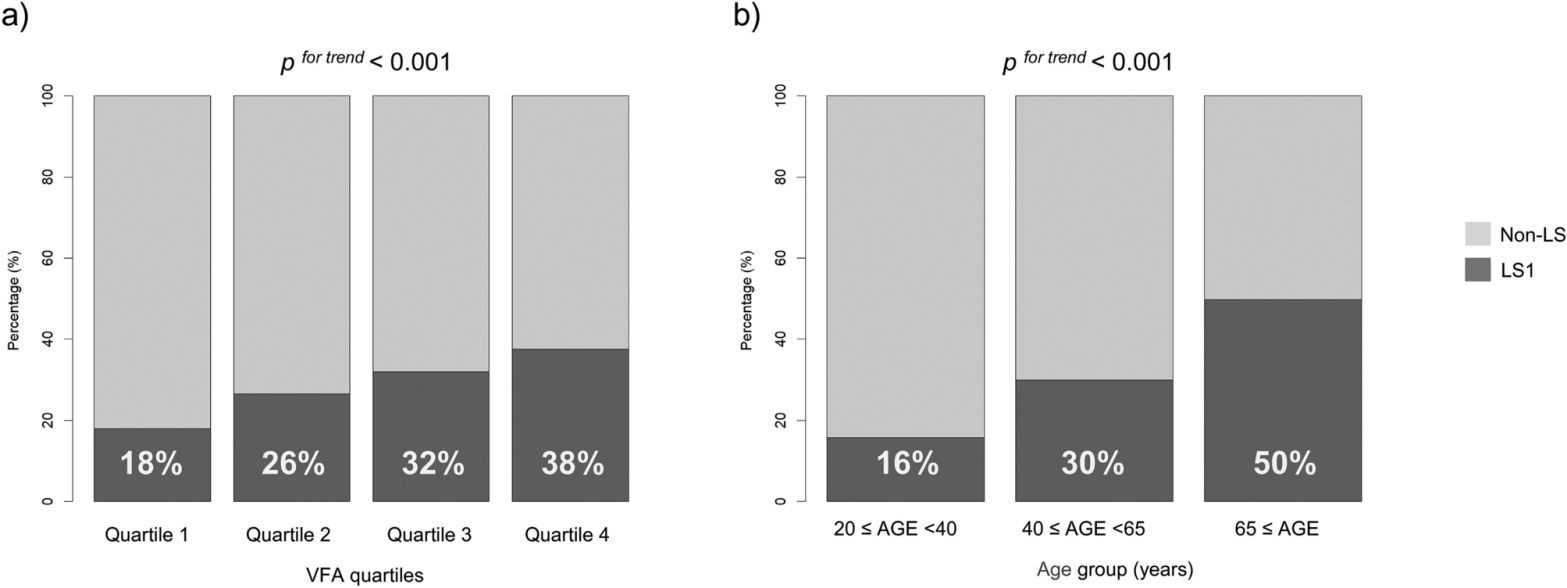
Proportion of patients with stage 1 locomotive syndrome. (**a**) Proportion of patients with LS1 divided into quartiles based on VFA. (**b**) Proportions of patients with LS1 in the young, middle-aged, and older groups.

The participants in the LS1 group had a higher VFA (90.8 vs. 75.5 cm^2^, *p* < 0.001), a higher BMI (23.6 vs. 22.4 kg/m^2^, *p* < 0.001), an older age (54.9 vs. 45.3 years, *p* < 0.001), and a lower height (160.9 vs. 163.3 cm, *p* < 0.001) than those in the non-LS group. The proportion of LS1 divided into quartiles based on VFA quartiles was 18%, 26%, 32%, and 38% in quartiles 1, 2, 3, and 4, respectively (Fig. 2a), indicating a positive association between the VFA quartiles and the proportion of LS1 (*p* for trend < 0.001). The proportion of LS1 in the young adult (20 ≤ age < 40 years), middle-aged (40 ≤ age < 65 years), and older (65 years ≤ age) groups was 16%, 30%, and 50%, respectively (Fig. 2b). These results indicated that the higher the age group, the higher the proportion of LS1 (*p* for trend < 0.001).

**Fig. 3:**
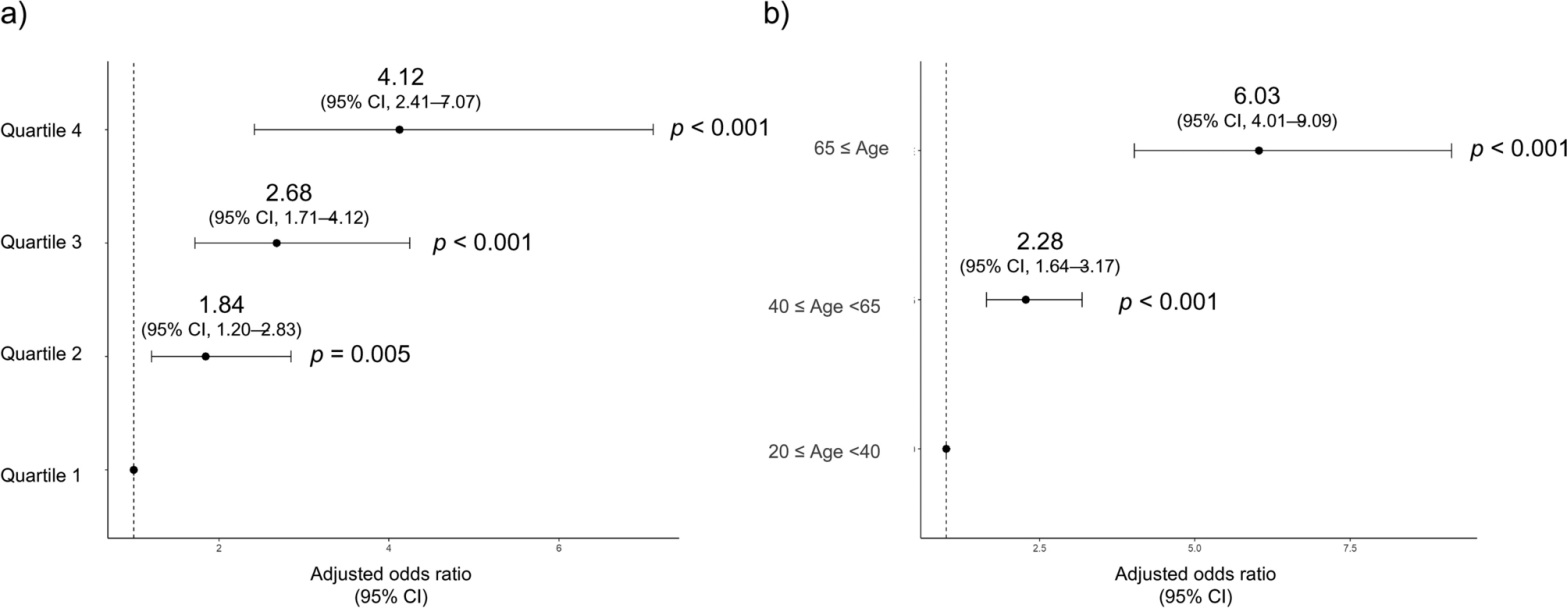
Adjusted odds ratio (OR) for stage 1 locomotive syndrome. (**a**) Visceral fat area quartiles. (**b**) Age groups. Circles indicate point estimates compared with the reference. The width of the horizontal lines represents the 95% confidence intervals (CIs) for each explanatory variable.

The multiple logistic regression analyses across the VFA quartiles revealed a significant association between VFA and LS1 prevalence: quartiles 2 (OR, 1.84; 95% CI, 1.20–2.83; *p* = 0.005), 3 (OR, 2.68; 95% CI, 1.71–4.12; *p* < 0.001), and 4 (OR, 4.12; 95% CI, 2.41–7.07; *p* < 0.001), compared with quartile 1 (Fig. 3a).

The multiple logistic analyses across age groups revealed a significant association between age and LS1 prevalence: middle-aged (OR, 2.28; 95% CI, 1.64–3.17; *p* < 0.001) and older groups (OR, 6.03; 95% CI, 4.01–9.09; *p* < 0.001), compared with the young adult group (Fig. 3b).

**Fig. 4:**
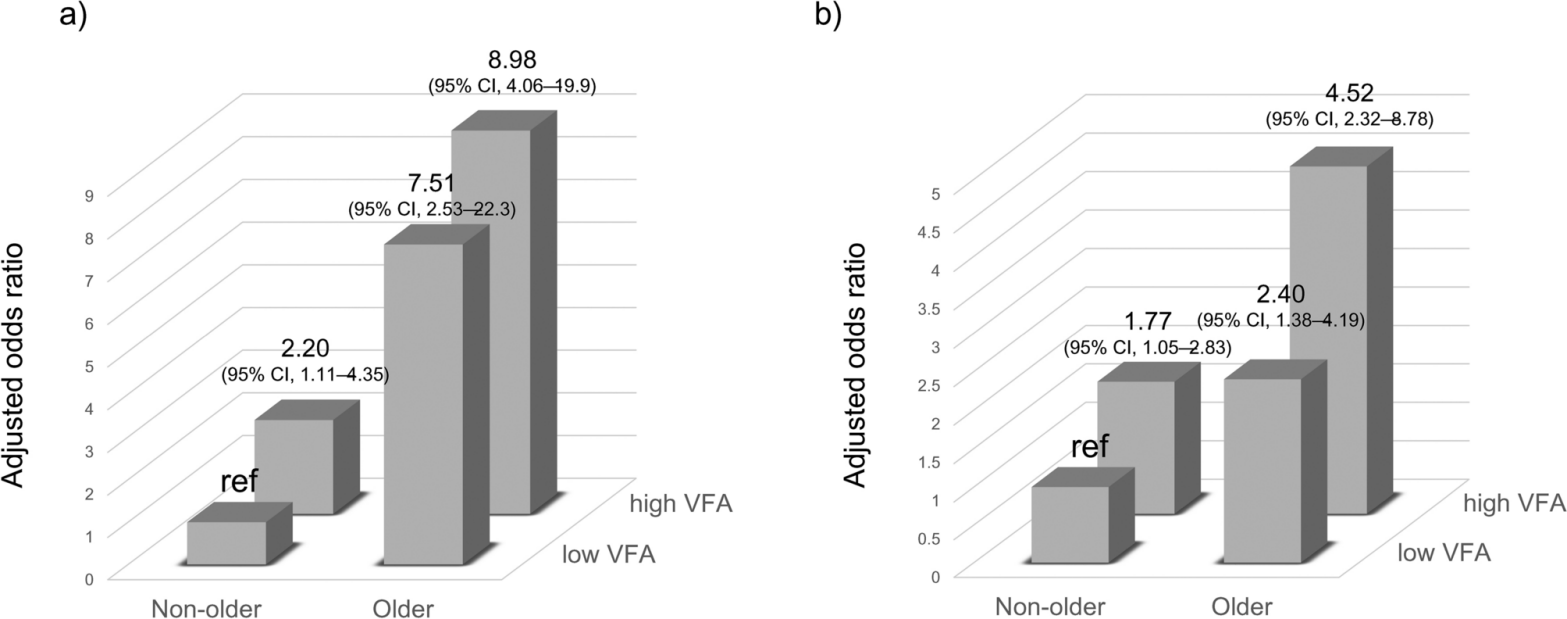
Adjusted odds ratio (OR) for stage 1 locomotive syndrome among the four groups stratified by sex. (**a**) Male. (**b**) Female. Adjusted odds ratios of the high VFA (73 < VFA) and non-older group (age < 65 years), low VFA (VFA ≤ 73) and older group (65 ≤ age years), or high VFA (73 < VFA) and older group (65 years ≤ age), compared with the reference (ref, low VFA (VFA ≤ 73) and non-older group (age < 65 years)), are shown. Adjustment variables included body mass index, skeletal muscle mass index, T-score, exercise habits, smoking status, and alcohol consumption.

The multiple logistic analysis stratified by sex showed a significant association between the LS1 prevalence and four groups in both male and female individuals: in male individuals, high VFA and non-older (OR, 2.20; 95% CI, 1.11–4.35; *p* = 0.024), low VFA and older (OR, 7.51; 95% CI, 2.53–22.3; *p* < 0.001), and high VFA and older groups (OR, 8.98; 95% CI, 4.06–19.9; *p* < 0.001), compared with the low VFA and non-older group; in female individuals, high VFA and non-older (OR, 1.77; 95% CI, 1.05–2.83; *p* = 0.032), low VFA and older (OR, 2.40; 95% CI, 1.38–4.19; *p* = 0.002), and high VFA and older groups (OR, 4.52; 95% CI, 2.32–8.78; *p* < 0.001), compared with the low VFA and non-older group. The analysis excluded sex as an explanatory variable, while the other variables remained the same as previously mentioned (Fig. 4).

The multiple logistic analysis of the four groups stratified by median VFA and age showed a significant association between the LS1 prevalence and four groups: high VFA and non-older group (OR, 1.87; 95% CI, 1.28–2.72; *p* = 0.001), low VFA and older group (OR, 3.16; 95% CI, 1.94–5.14; *p* < 0.001), and high VFA and older group (OR, 6.43; 95% CI, 3.98–10.4; *p* < 0.001), compared with the low VFA and non-older group (Supplementary Fig. S1).

## Discussion

In the present study, we investigated the association between LS1 and VFA in Japanese community-dwelling adults aged 20–85 years. Our findings demonstrated that VFA and age were significantly associated with LS1 even after adjusting for several factors. This study also suggested that VFA was cumulatively associated with LS1 in relation to age in both the older and non-older groups. Although a previous study focused on older individuals, this is the first study to investigate the association between LS1 and VFA, in addition to clarifying the involvement of VFA in LS1 across age groups.

Our findings showed a positive association between LS prevalence and VFA, wherein VFA showed a significant association with LS1 (Fig. 3a). Age was also significantly associated with LS1 (Fig. 3b). Therefore, we investigated the interaction of VFA and age using multiple logistic analyses of the four groups divided by the VFA median (Fig. S1). The OR for the high VFA and older group was significantly higher than that of the low VFA and non-older group and was the highest (6.43) among the four groups. Furthermore, the OR for the high VFA and non-older group was 1.87 and that for the low VFA and older group was 3.16. These findings suggest that the involvement of VFA in LS1 is stronger in older individuals than that in non-older individuals.

These findings are consistent with those of a previous study in older individuals aged ≥ 85 years, which showed a significant association between VFA with mobility disability equivalent to LS3^27^. Furthermore, studies have shown that VFA in Japanese adults gradually increases with age^35,36^, particularly, from 40 to 79 years of age in 42.9% of men and 65.5% of women^29^. It has also been reported that the age-dependent increases in VFA and inflammatory cytokines, such as tumour necrosis factor-α and interleukin-6, might result in skeletal muscle mass loss^29^. The production of these cytokines is facilitated by the increase in visceral adipose tissue proportion, and their levels increase with age^37,38^. This finding could explain the varying effects of VFA between older and non-older individuals. Furthermore, the present study showed that VFA was associated with LS1, particularly in the early stages of LS, in both age groups.

In the non-older group, the VFA and LS1 prevalence ratio was 15.8% in the young adult group (20 ≤ age < 40) and 29.9% in the middle-aged group (40 years ≤ age < 65). The OR for the high VFA and non-older group was lower (1.87) than that of the low VFA and non-older group (3.16), indicating the involvement of ageing in LS1, aligning with previous findings^25^. A comparison of the ORs of the low VFA and older and the high VFA and non-older groups indicated that VFA may cumulatively affect LS1 with age in both age groups. Therefore, managing VFA through diet and exercise is crucial for preventing LS1 in both older and non-older individuals. However, the proportion of LS1 in each age group in our study was generally lower than that in the ROAD study (e.g. 21.7% in male individuals and 25% in female individuals, aged < 40 years)^25^. Unlike the ROAD study, which included diverse areas such as rural mountainous and urban and seaside areas, our study focused on rural mountainous area residents, many of whom were engaged in agriculture, possibly contributing to the difference in prevalence.

In our study, the LS1 group was significantly older and shorter and had a higher BMI than the non-LS group, generally consistent with those of previous studies^9,25,26^. However, no significant differences in lifestyle habits related to LS have been reported^8,19^.

A strength of this study is the inclusion of a wide range of age groups with VFA measured using the abdominal bioimpedance method and LS assessed using the clinical criteria. Nonetheless, this study has some limitations. First, this was a cross-sectional study and not a longitudinal cohort study, and we could not assess high VFA as a risk factor for the future prevalence of LS1. Second, this study included participants from a particular race or region; therefore, the findings need validation across different races or regions. Third, this study did not account for the effects of medication or treatment history. Fourth, the age distribution in this study differs from that of the overall age distribution in Japan. Finally, self-reported lifestyle habits and physical activity data may also be subject to recall bias.

## Conclusions

VFA and age are significantly associated with LS1. Additionally, VFA is cumulatively associated with LS1 in relation to age in both the older and non-older groups. Our findings indicate that the management of VFA by diet and exercise is crucial for not only older but also non-older individuals to prevent LS1.

## Supplementary Information

Supplementary Information 1.

Supplementary Information 2.

## Data Availability

All data analysed during this study are available on request from the Hirosaki University COI Program Institutional Data Access/Ethics Committee (contact via e-mail: coi@hirosaki-u.ac.jp) for researchers who meet the criteria for access to the data. Researchers must be approved by the research ethics review board at the organisations of their affiliations. The data cannot be shared publicly because of ethical concerns.
