# Supplementary material for: Association of visceral fat area with early-stage locomotive syndrome across various age groups: a cross-sectional study
Source: Sci Rep. 2024 Oct 26;14:25498. doi: 10.1038/s41598-024-76478-8 (PMC11513122; doi:10.1038/s41598-024-76478-8)
Supplement: Supplementary file 1 — Supplementary Information 1. [file 41598_2024_76478_MOESM1_ESM.docx]

**Supplementary Figure S1**. Adjusted odds ratio (OR) for stage 1 locomotive syndrome among the four groups. Adjusted odds ratios of the high VFA (> 73) and non-older group (< 65 years), low VFA (≤ 73) and older group (≥ 65 years), or high VFA and older group, compared with the reference (ref, low VFA and non-older group (age < 65 years)), are shown.
