# Supplementary material for: Association of visceral fat area with early-stage locomotive syndrome across various age groups: a cross-sectional study
Source: Sci Rep. 2024 Oct 26;14:25498. doi: 10.1038/s41598-024-76478-8 (PMC11513122; doi:10.1038/s41598-024-76478-8)
Supplement: Supplementary file 2 — Supplementary Information 2. [file 41598_2024_76478_MOESM2_ESM.pptx]

## Slide 1
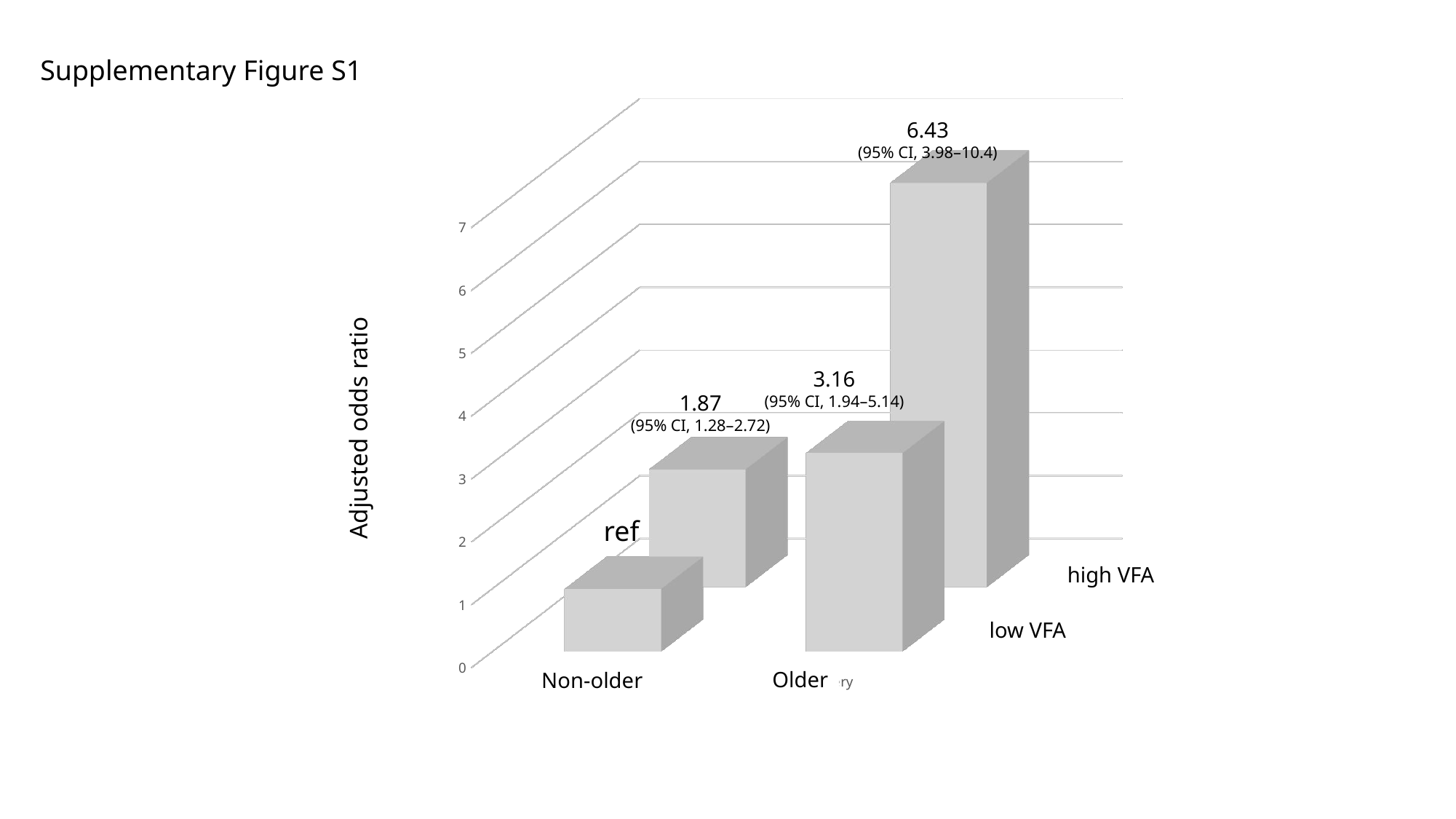

Supplementary Figure S1
[unsupported chart]
6.43
(95% CI, 3.98–10.4)
3.16
(95% CI, 1.94–5.14)
1.87
(95% CI, 1.28–2.72)
Adjusted odds ratio
ref
high VFA
low VFA
Older
Non-older
